# Differential temperature sensitivity of synaptic and firing processes in a neural mass model of epileptic discharges explains heterogeneous response of experimental epilepsy to focal brain cooling

**DOI:** 10.1371/journal.pcbi.1005736

**Published:** 2017-10-05

**Authors:** Jaymar Soriano, Takatomi Kubo, Takao Inoue, Hiroyuki Kida, Toshitaka Yamakawa, Michiyasu Suzuki, Kazushi Ikeda

**Affiliations:** 1 Mathematical Informatics Laboratory, Graduate School of Information Science, Nara Institute of Science and Technology, Ikoma, Japan; 2 Department of Computer Science, University of the Philippines - Diliman, Quezon City, Philippines; 3 Department of Neurosurgery, Yamaguchi University Graduate School of Medicine, Ube, Japan; 4 Department of Physiology, Yamaguchi University Graduate School of Medicine, Ube, Japan; 5 Organization for Innovation and Excellence, Kumamoto University, Kumamoto, Japan; Medical College of Wisconsin, UNITED STATES

## Abstract

Experiments with drug-induced epilepsy in rat brains and epileptic human brain region reveal that focal cooling can suppress epileptic discharges without affecting the brain’s normal neurological function. Findings suggest a viable treatment for intractable epilepsy cases via an implantable cooling device. However, precise mechanisms by which cooling suppresses epileptic discharges are still not clearly understood. Cooling experiments *in vitro* presented evidence of reduction in neurotransmitter release from presynaptic terminals and loss of dendritic spines at post-synaptic terminals offering a possible synaptic mechanism. We show that termination of epileptic discharges is possible by introducing a homogeneous temperature factor in a neural mass model which attenuates the post-synaptic impulse responses of the neuronal populations. This result however may be expected since such attenuation leads to reduced post-synaptic potential and when the effect on inhibitory interneurons is less than on excitatory interneurons, frequency of firing of pyramidal cells is consequently reduced. While this is observed in cooling experiments *in vitro*, experiments *in vivo* exhibit persistent discharges during cooling but suppressed in magnitude. This leads us to conjecture that reduction in the frequency of discharges may be compensated through intrinsic excitability mechanisms. Such compensatory mechanism is modelled using a reciprocal temperature factor in the firing response function in the neural mass model. We demonstrate that the complete model can reproduce attenuation of both magnitude and frequency of epileptic discharges during cooling. The compensatory mechanism suggests that cooling lowers the average and the variance of the distribution of threshold potential of firing across the population. Bifurcation study with respect to the temperature parameters of the model reveals how heterogeneous response of epileptic discharges to cooling (termination or suppression only) is exhibited. Possibility of differential temperature effects on post-synaptic potential generation of different populations is also explored.

## Author summary

Focal cooling of the epileptic brain region has been shown to consistently suppress epileptic activity and it is hoped that this treatment can be developed in the future into an implantable cooling device. However, it is still not clearly understood how cooling suppresses epileptic activity. This study uses a computational approach to identify and investigate possible mechanisms. First, we used a neural mass model to reproduce epileptic discharge activity. Next, we simulate the effect of cooling by introducing temperature dependence in the model. Based from evidences reported from *in vitro* and *in vivo* studies, we formulated two temperature-dependent mechanisms that can reproduce the effect of cooling on the epileptic discharge activity. Parameter estimation of the model was performed using EEG recordings of focal brain cooling experiments with rats *in vivo*. Our model involves a synaptic mechanism that results in a reduced frequency of discharges and an intrinsic excitability mechanism that compensates such reduction in frequency of discharges resulting in persistent discharges during cooling but suppressed in magnitude. The temperature dependence is in the form of Q_10_ temperature coefficients which determine whether suppression or termination of epileptic discharges can be achieved.

## Introduction

The World Health Organization identifies epilepsy as one of the most common neurological diseases affecting approximately 50 million people across all ages across the world [1]. According to the International League Against Epilepsy, a patient has epilepsy if he has had a seizure and his brain activity demonstrates a pathologic and enduring predisposition to have recurrent seizures [2]. Because of the risks involved with unanticipated seizures, treatment of the disease is required to improve long-term quality-of-life of the patients. Antiepileptic drugs such as anticonvulsants are usually given as first line treatment after being diagnosed with epilepsy. Pharmaceutical researches continually seek antiepileptic drugs that are more effective and have less side effects [3, 4]. However, 20%-40% of patients diagnosed with epilepsy are found refractory to antiepileptic drug treatment [5, 6]. Thus, alternative treatments are still being sought after [7, 8]. Surgical treatment is done by performing a resection of the epileptic foci of the brain. Absolute remission however is not guaranteed, let alone possibilities of unintended outcomes since the method is largely invasive [9, 10]. Although the success rate of surgical treatment is high, limitation in indication and cost significantly hinder intractable epilepsy patients in acquiring it. Another increasingly attractive treatment option involves electrical neurostimulation of specific neural region such as vagus nerve stimulation and deep brain stimulation [11, 12].

In the previous decade, focal cooling of the epileptic brain area has been pursued as an alternative therapeutic treatment for epilepsy and other seizure-inducing brain injuries [13–15]. Studies in animals have shown that reversible cooling to a temperature as low as 15°C using an implantable cooling device is able to terminate epileptic discharges without affecting the normal brain tissue [16–19]. Earlier experiments even noted that focal cooling of the cortex for one hour above 0°C did not induce any irreversible histological change or motor dysfunction [20]. Focal cooling at 25°C was also demonstrated to suppress epileptic discharges in a human brain [21]. Epileptic seizures arising from post-traumatic brain injuries were also shown to be suppressed and can be further prevented by moderately cooling the brain down by a temperature reduction of 2°C [22]. In other studies, focal brain cooling has found potential use for treatment of other brain diseases such as ischaemia, stroke, and neonatal encephalopathy [23–26]. The ultimate goal especially for epilepsy studies is to develop a technique for epileptic seizure suppression by a temperature control, when detected, via an implantable cooling device as a solution for thermal neuromodulation. This is feasible if we have precise knowledge of how temperature can suppress or terminate seizures. While temperature effects on physiological properties of animal neurons have been well-studied *in vitro* [27–31], mechanisms of how cooling suppresses epileptic discharges especially *in vivo* are still not clearly understood.

In this study, we aim to identify prospective mechanisms and investigate them using a computational modelling approach. Neural mass models have been widely utilized to study brain activities [32–37] and gain relevant physiological insights from them. The model introduced by Wendling et al. [34] in particular was shown to produce different types of brain activities similar to intracranial EEG recordings. We explore prospective mechanisms of cooling on epileptic discharges by introducing temperature dependence in the neural mass model of Wendling et al. in light of findings observed in *in vitro* and *in vivo* experiments published in literature. In particular, changes in synaptic dynamics were reported from *in vitro* cooling experiments such as reduction in the efficacy of neurotransmitter vesicle release [38], loss of dendritic spines [39] and reduced glutamate concentrations [40, 41], suggesting a possible synaptic mechanism. A recent study with patients with intractable epilepsy also reports reduced extracellular glutamate and GABA concentrations during focal brain cooling [42]. We then formulated temperature dependence in our chosen neural mass model by introducing a temperature factor in the post-synaptic impulse response function. Parameter estimation of the model is performed using EEG recordings from *in vivo* cooling experiments on an animal model of epilepsy. Although the model is able to reproduce termination of epileptic discharges reported in *in vitro* studies [43, 44], the results of modeling our experimental data (*in vivo*) reveal that this synaptic mechanism is not sufficient to explain epileptic discharges that are persistent during cooling although suppressed in magnitude. We propose that another mechanism is required to compensate the effect of this synaptic mechanism to be able to reproduce observed suppression of epileptic discharges during cooling in terms of reduction in both frequency and magnitude of discharges. We suggest some biological plausibility of this compensatory mechanism based from published results from cooling experiments. The temperature dependence is in the form of a temperature coefficient (Q_10_) which represents the factor by which the rate of a process increases for every ten-degree rise in the temperature at which it takes place [45]. In this study, the Q_10_ values determine whether suppression or termination of epileptic discharges can be achieved. Such heterogeneous response of epileptic discharge activity to cooling is revealed by bifurcation patterns with respect to the temperature parameters of the model.

## Materials and methods

### Ethics statement

All experiments were performed according to the Guidelines for Animal Experimentation of Yamaguchi University School of Medicine. The animals were anesthetized with urethane (1.25 g/kg, i.p.). Lidocaine, a local anesthetic, was applied at pressure points and around the area of surgery.

### Focal brain cooling experiment

Focal brain cooling experiments were performed at Yamaguchi University School of Medicine. In this study, we utilized their data for parameter estimation of our model. Details of the experiments can be found in [46]. Briefly, anaesthetized male Sprague-Dawley rats were induced with epilepsy using Penicillin G potassium. Continuous EEG recordings of the epilepsy-induced region of the brain were made before and during cooling. An Ag/AgCl electrode for recording EEGs (Unique Medical Co., Fukuoka, Japan) was positioned stereotactically 2 mm below the cortical surface at the left sensorimotor cortex just beneath the cooling device. Five different rat experiments each were done at cooling temperatures 25°C, 20°C, and 15°C. To remove high frequency components and also match the represented frequencies in the model, the raw recordings underwent a 40-Hz low-pass filter using a fifth order Butterworth filter in Matlab. One-minute steady-state intervals before and during cooling were identified by an expert and were taken from the filtered data for the study. For the model estimation procedure, first, the data is further downsampled to 2kHz corresponding to a step size of 0.5 ms in the simulation. Next, both the downsampled data and the simulated EEG are normalized by dividing by their respective standard deviations of activity before cooling, thus, they are reported in arbitrary units (au) unless otherwise stated.

**Fig 1:**
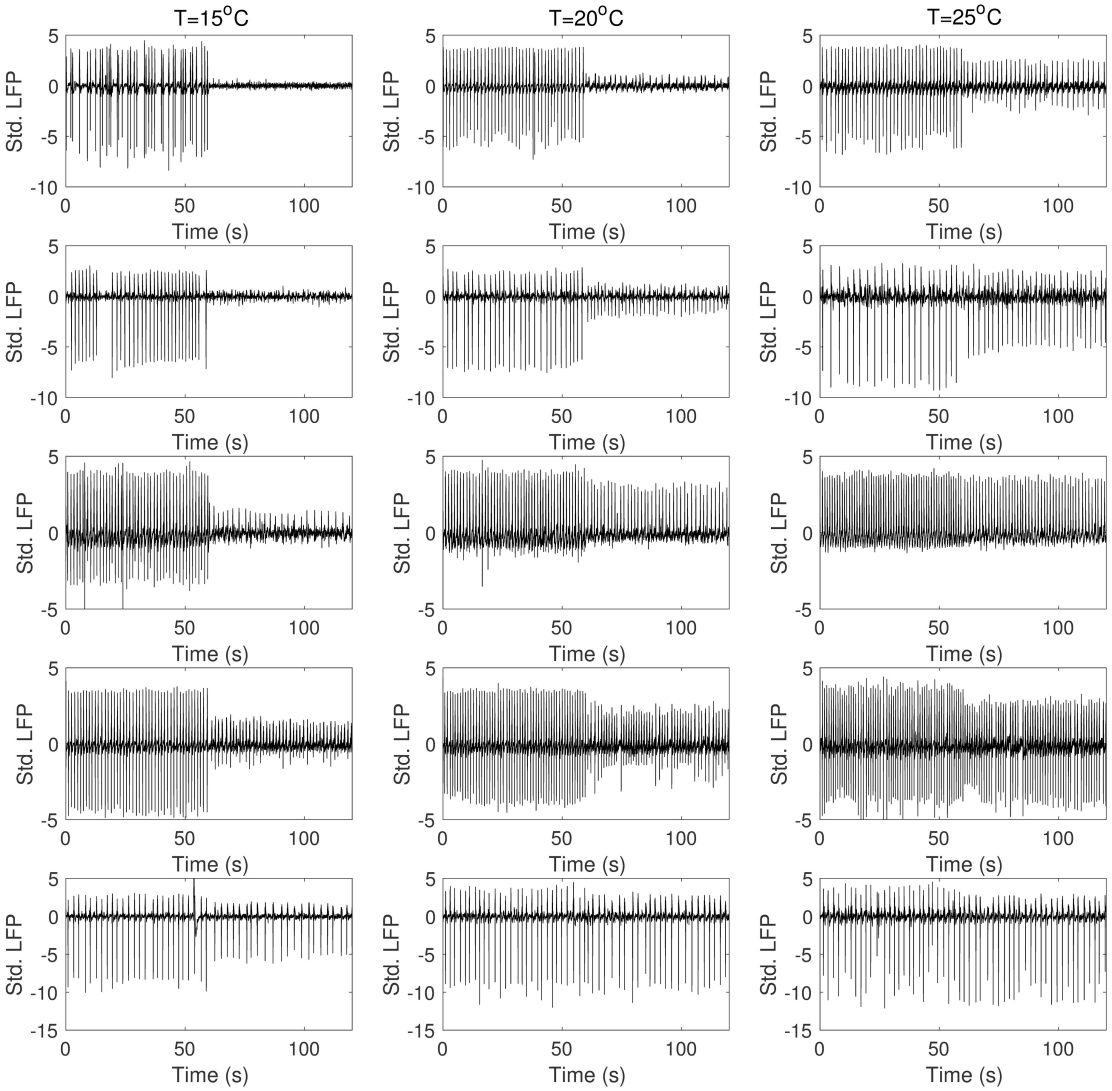
Experimental data. Concatenated one-minute steady state activities before (0s-60s) and during (60s-120s) cooling identified by an expert. (From top to bottom: rat 1 to rat 5.)

**Fig 2:**
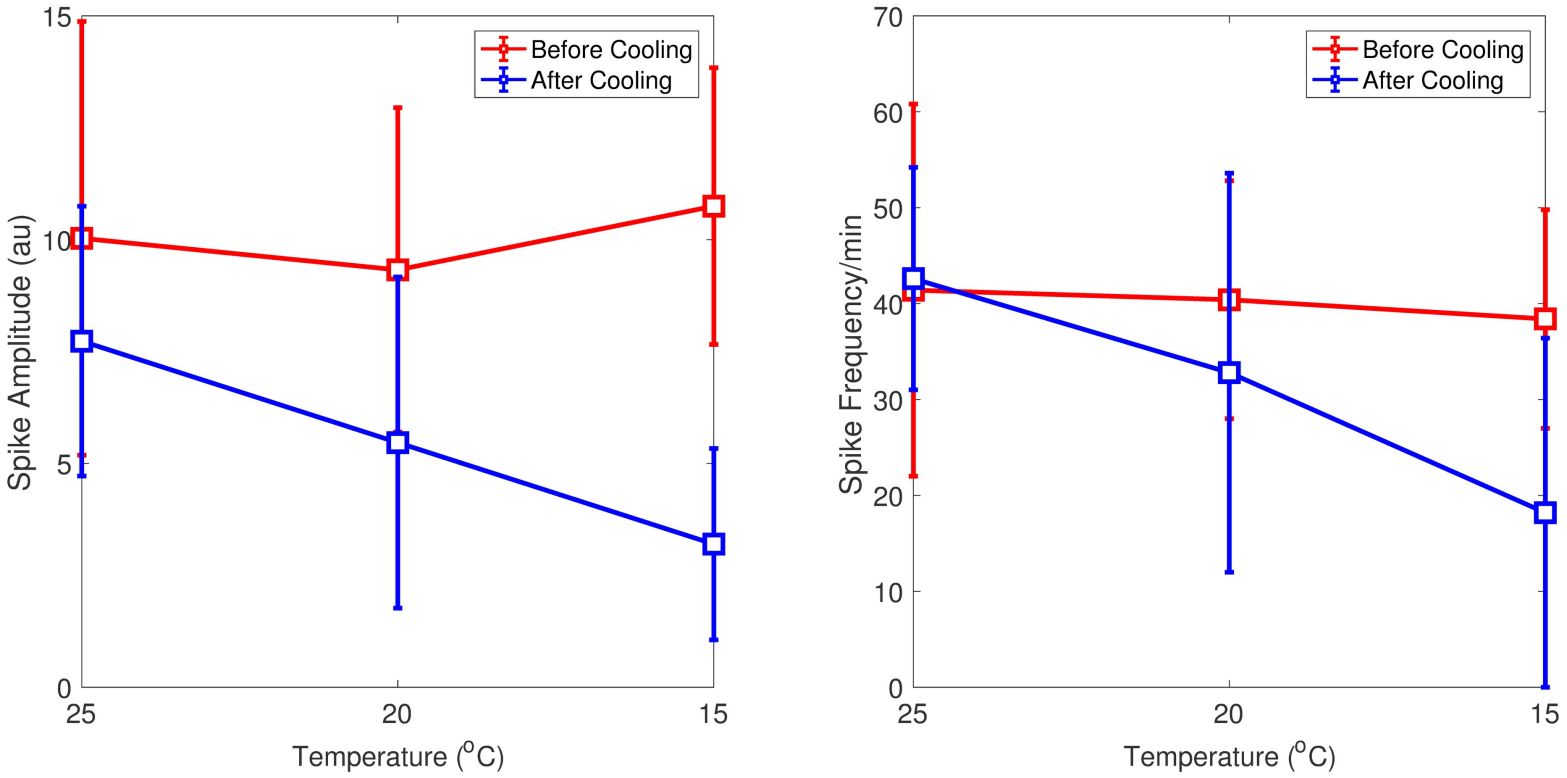
Magnitude and frequency of epileptic discharges before and during cooling. Magnitude of epileptic discharges before cooling (red) decreased during focal cooling (blue) in all cases (left). Frequency of epileptic discharges also decreased during cooling in most cases (right) with slight increases seen in some cases (Fig 1). Error bars indicate minimum and maximum observations from five rats.

Fig 1 shows a summary of the preprocessed data in which we concatenated one-minute steady state activities before and during cooling. Suppression of epileptic discharges during cooling was observed especially with 15°C cooling temperature (Fig 1). Epileptic discharges were suppressed in terms of magnitude (lower magnitude during cooling) in all cases. In most cases, frequency of epileptic discharges is lower during cooling although slightly higher in some cases. The average magnitude and frequency of epileptic discharges before and during cooling are summarized in Fig 2 with error bars indicating minimum and maximum values from five rats. In general, we can say that epileptic discharges are suppressed during focal cooling at all three cooling temperatures. Surprisingly, significant termination of epileptic discharges was observed only in two out of five rats with 15°C cooling temperature compared to most *in vitro* recordings reported in literature; epileptic discharges were generally persistent during cooling from these *in vivo* recordings.

### Neural mass model

Different intracranial EEG activities such as spike-wave discharges and low-voltage high-frequency activity, have been widely explained using neural mass models—a class of models based on a mean-field approximation of the activity of a population of neurons. Neural mass models involve two major processes described by two functions: a firing response function and a post-synaptic impulse response function. The firing response function approximates the average firing rate of a population in response to an average input potential (the average membrane potential of the population). Assuming a unimodal distribution of threshold potentials, the firing response function of a population of neurons can be described by a sigmoid function [47] given by

$$\begin{array}{c} S\left(v\right)=\frac{2e_{0}}{1+e^{\left(\frac{v_{th}-v}{\sigma_{th}}\right)}}, \end{array}$$ (1)

where *v*_*th*_ is the average threshold potential at which the population fires at half the maximum firing rate *e*_0_. The steepness of the sigmoid curve 1/*σ*_*th*_ is inversely related to the variability in thresholds of excitation of neurons in the population [47]. On the other hand, the average post-synaptic potential (PSP) input of a neuronal population to other populations to which it provides excitation or inhibition is given by the convolution of the post-synaptic impulse response function *h*(*t*) of the population and its average firing rate *u*(*t*). Originally, the post-synaptic impulse response function is modelled using a sum of two exponentials [32] as compared from experimental data but was later simplified to

$$\begin{array}{c} h_{X}\left(t\right)=G_{X}g_{X}te^{-g_{X}t};\;t\geq 0, \end{array}$$ (2)

where *G*_*X*_ is the average post-synaptic gain and *g*_*X*_ is the reciprocal of the average synaptic time constant of population *X*. Finally, the convolution *v*_*X*_(*t*) = *h*_*X*_(*t*) * *u*(*t*) is equivalent to the solution of the following second-order differential equation using Green’s Formula [48]:

$$\begin{array}{c} v_{X}^{\prime \prime }+2g_{X}v_{X}^{\prime }+g^{2}v=G_{X}g_{X}u. \end{array}$$ (3)

The primary cell population also receives additional noisy input from subcortical afferents or other neural masses which makes the differential equation stochastic. Such can be solved numerically using stochastic methods such as Euler-Maruyama scheme. Finally, the average membrane potential of a population, which is the input to Eq (1), is taken as the weighted summation of the average post-synaptic potentials of the afferent populations (inhibitory populations have negative contribution). The weights are determined by the number of synaptic connections. The average membrane potential of the primary cell population is taken as representative of cortical EEG activity [32].

**Fig 3:**
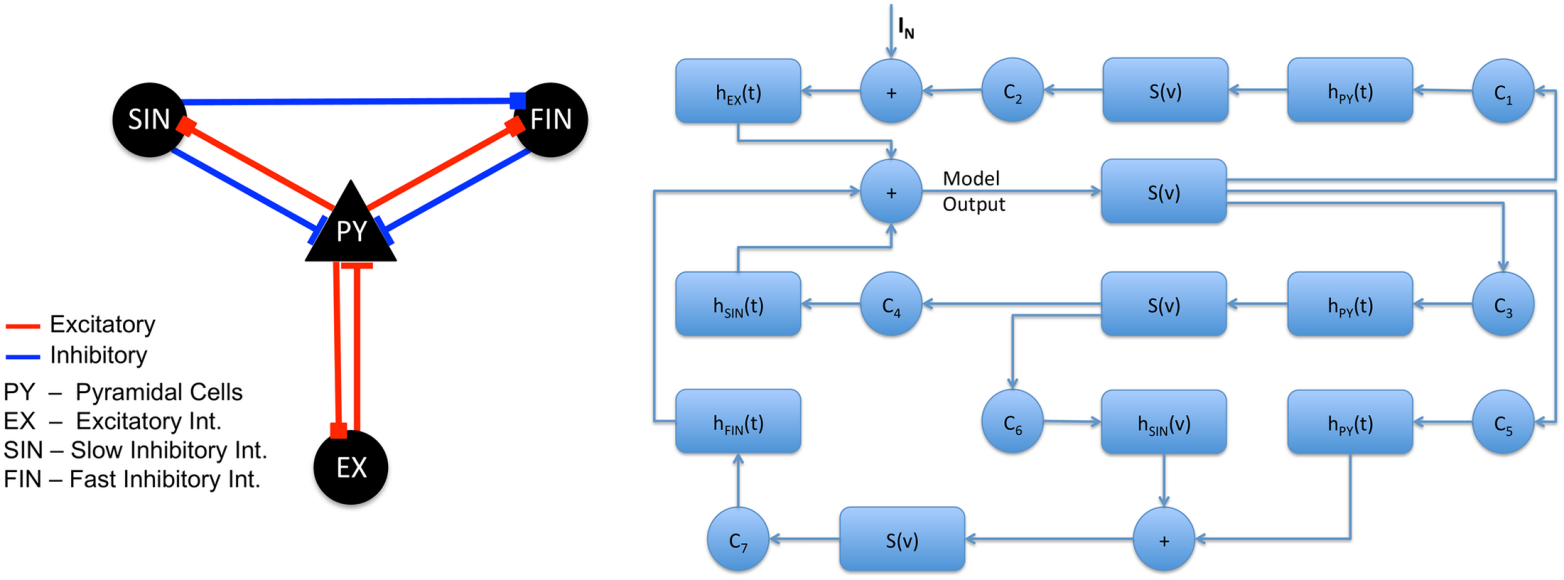
Neural mass model by Wendling et al. Block diagram of the model showing the interconnections among the neural populations (left) and the processes involved for each population (right).

Different neural mass models vary in terms of the types of neurons that comprise a population and the interconnections among the populations (feedback loops). Da Silva et al. [32] tried to explain alpha rhythm of brain activity by considering two populations: excitatory thalamocortical neurons as primary cell population and and a population of inhibitory interneurons. Jansen and Rit [33] extended this model using pyramidal cells as the primary excitatory neurons and two types of interneurons—excitatory and inhibitory. They also estimated the relations among the number of synaptic interconnections among the neuronal populations using animal records of cortical synapses found in literature. Wendling et al. [34] further differentiated slow and fast inhibitory interneurons based on the studies of [49, 50] based from hippocampal connections. In their model, slow inhibitory interneurons project to the dendrites while fast inhibitory interneurons project to the soma or near the soma of pyramidal cells. Moreover, slow inhibitory interneurons provide inhibition to fast inhibitory interneurons. Although the model was patterned after neuronal connections in hippocampus, similar architecture has been seen in the neocortex (see [51] for an extensive review). The block diagram of the model is shown in Fig 3. The parameters of the model are summarized in Table 1 together with the standard values adopted in this study.

**Table 1:** Description of model parameters and adopted values.

Paramater	Description	Values adopted in this study
*G*	Average synaptic gain (mV)	*G*_*PY*_ = *G*_*EX*_ = 5.0; *G*_*SIN*_ ∈ [25.0, 31.0]; *G*_*FIN*_ ∈ [85.0, 105.0]
*g*	Reciprocal of average synaptic time constant (Hz)	*g*_*PY*_ = *g*_*EX*_ = 100; *g*_*SIN*_ = 50; *g*_*FIN*_ = 500
*C*	Number of synaptic connections	*C*_*PY* → *EX*_ = 135;
		*C*_*EX* → *PY*_ = 0.8*C*_*PY* → *EX*_;
		*C*_*PY* → *SIN*_ = 0.25*C*_*PY* → *EX*_
		*C*_*PY* → *FIN*_ = 0.3*C*_*PY* → *EX*_;
		*C*_*SIN* → *PY*_ = 0.25*C*_*PY* → *EX*_
		*C*_*FIN* → *PY*_ = 0.8*C*_*PY* → *EX*_;
		*C*_*SIN* → *FIN*_ = 0.1*C*_*PY* → *EX*_
*v*_*th*_	Average threshold potential of firing (mV)	6.0
2*e*_0_	Maximum firing rate (Hz)	5.0
1/*σ*_*th*_	Steepness of sigmoid curve	0.56
*I*_*N*_	Subcortical input firing rate	N(μ=90,σ=30)

Wendling et al. showed that their model is able to capture different brain activities observed in intracranial EEG recordings. By fixing the value of average excitatory synaptic gain, an activity map (Figure 4 of [34]) shows regions of different brain activities by varying the average synaptic gains of slow and fast inhibitory neuronal populations. They used their model to explain that fast epileptic activity can arise due to impaired GABAergic inhibition by slow inhibitory interneurons. They demonstrated this by estimating average synaptic gains in the model from intracranial EEG recordings of temporal lobe epilepsy (Figure 5 and 6 of [34]). In this study, we used the same model and show that it strongly captures the discharge activity of the animal model of epilepsy used in the experiments.

### Model formulation

In this study, we try to explain how cooling works in suppressing epileptic discharges by introducing temperature dependence in the neural mass model of Wendling et al. particularly for epileptic discharges. Our formulation starts with reduction in concentration of neurotransmitters as reported in *in vitro* studies. We model this effect as an attenuation factor in the post-synaptic impulse response function particularly the average synaptic gain variable. Specifically, we assume a temperature dependence in terms of a Q_10_ factor as follows:

$$\begin{array}{c} h_{X}\left(t\right)=Q_{10,syn}^{(T-T_{0})/10}G_{X}g_{X}te^{-g_{X}t};\;t\geq 0. \end{array}$$ (4)

**Fig 4:**
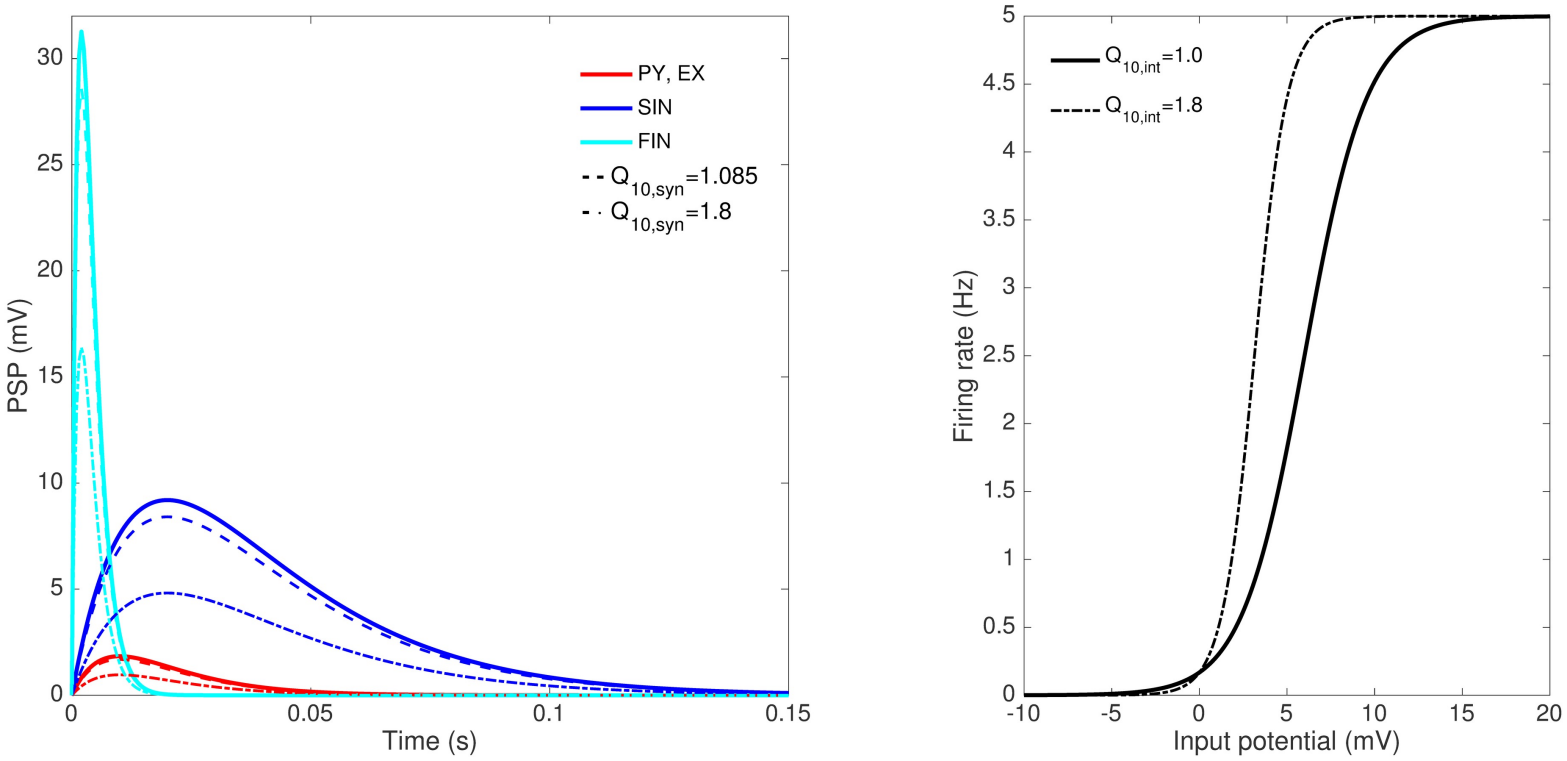
Response functions and the effect of Q_10_. Different populations of neurons have different post-synaptic impulse response (solid lines, left) but are assumed to have the same firing response (solid line, right). *Q*_10,*syn*_ scales down the post-synaptic impulse response curves (broken lines, left) while *Q*_10,*int*_ changes the properties of the firing response curve (broken line, right).

**Fig 5:**
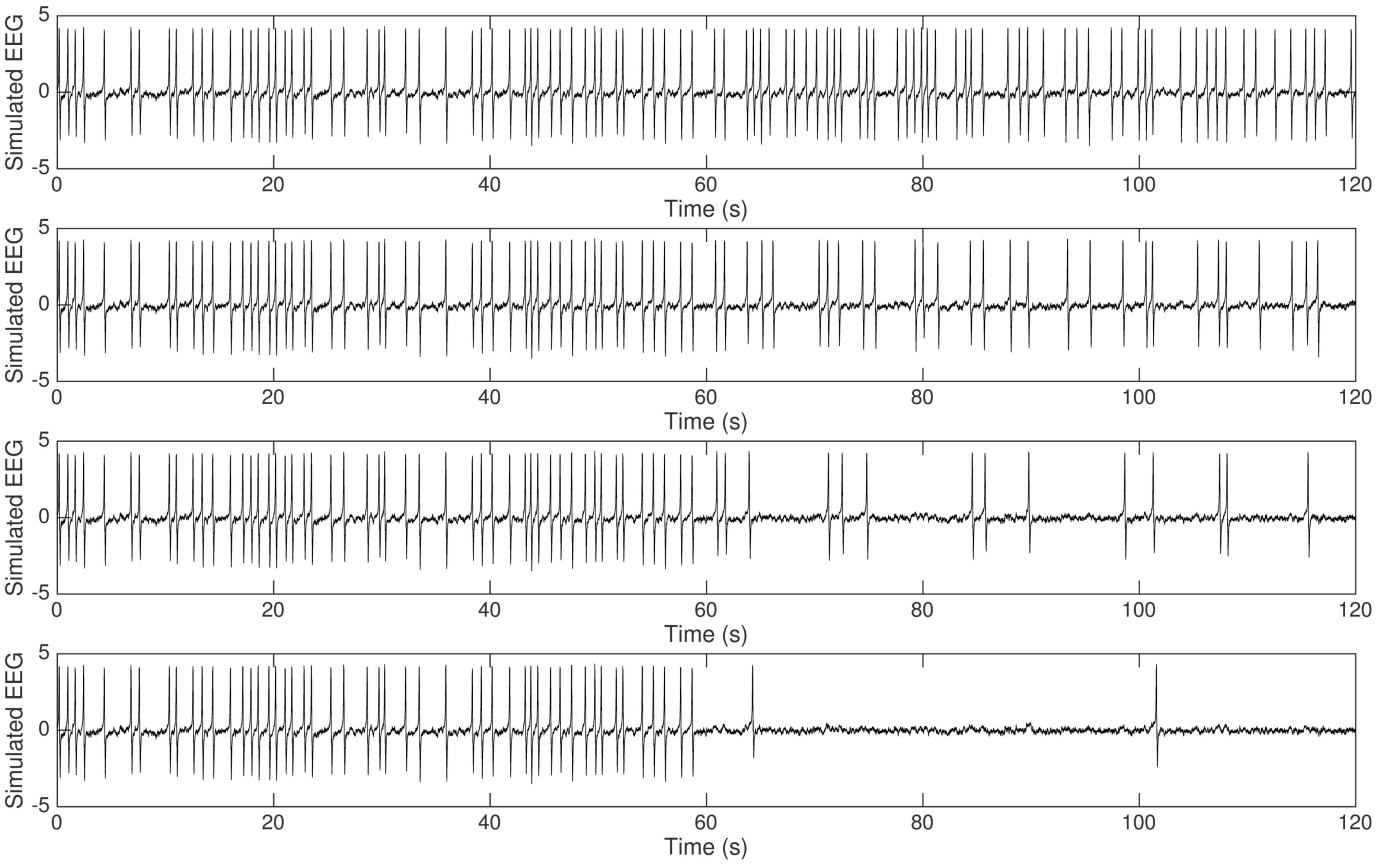
Effect of *Q*_10,*syn*_. As *Q*_10,*syn*_ is increased from unity, frequency of epileptic discharges during cooling (60 s–120 s) becomes less until complete termination. (From top to bottom: *Q*_10,*syn*_ = 1.0, 1.007, 1.013, 1.085.)

Here, *T*_0_ is the baseline temperature which is 31°C in the experiments. This temperature dependence attenuates the average synaptic gain and thus reduces the average PSP (Fig 4) which makes up the average membrane potential of the population to which it provides excitation or inhibition. For excitatory and slow inhibitory interneurons, their average membrane potentials are solely contributed by the average PSP from pyramidal cell population, thus, are also attenuated and consequently yield reduced firing frequency. For the pyramidal cell population and fast inhibitory interneurons, negative inhibitory PSP contributes to their average membrane potential. If the weighted (in terms of synaptic connections) effect of temperature on inhibitory PSP is less than that on excitatory PSP, a net decrease in average membrane potential results. With the parameter values chosen in the model (Table 1), this is more likely the case. In Fig 5, we can see that as *Q*_10,*syn*_ is increased from unity, frequency of discharges during cooling is decreased until termination. However, the value of *Q*_10,*syn*_ at which termination is nearly achieved (*Q*_10,*syn*_ = 1.085) does not significantly attenuate PSP magnitude (Fig 4), consequently the magnitude of isolated discharges. In contrast, persistent discharges were observed during cooling in the experiments (Fig 1). These are suggestive that another mechanism is involved. To model persistent discharges during the cooling period, we conjecture that the reduction in the average frequency of firing caused by the first temperature dependence should be compensated. This can be achieved through the firing response function negating the effect of *Q*_10,*syn*_ (see Discussion). A second temperature dependence is thus put forward involving a reciprocal Q_10_ factor multiplied to the average membrane potential:

$$\begin{array}{c} S\left(v\right)=\frac{2e_{0}}{1+e^{\left(\frac{v_{th}-Q_{10,int}^{-(T-T_{0})/10}v}{\sigma_{th}}\right)}}. \end{array}$$ (5)

Fig 4 illustrates the effect of this temperature dependence in the original firing response curve. The modified firing response curve is translated to the left and has steeper slope. In summary, two temperature parameters are introduced in this study—*Q*_10,*syn*_ and *Q*_10,*int*_. The latter part of this study also looks at the possibility that *Q*_10,*syn*_ varies for different populations in their respective PSP generation.

### Parameter estimation

Since the cooling experiments were performed on five rats, model parameters were estimated per rat using three cooling temperatures. Modified from [52], the objective function involved in the estimation is given by

$$\begin{array}{c} J\left(\theta \right)=\sum \;E_{IDI}+E_{EffMag}+P\left(\theta \right), \end{array}$$ (6)

where *E*_*x*_ is the mean absolute percentage error (MAPE) of feature *x* computed as |*x*_*model*_ − *x*_*data*_| / |*x*_*data*_|. The summation is over the three cooling experiments per rat. The features used in the estimation are the average inter-dischrage interval (IDI) over the one-minute series and the effective magnitude (*EffMag*) of epileptic discharges. *IDI* is computed as

$$\begin{array}{c} IDI=\frac{1}{N_{D}}\;\sum_{i=1}^{N_{D}-1}\;t_{i+1}-t_{i}, \end{array}$$ (7)

where *t*_*i*_ is a time at which a discharge (exceeding three standard deviations of the activity) occurs, and *N*_*D*_ is the number of discharges within the one-minute activity. *EffMag*, on the other hand, is defined as

$$\begin{array}{c} EffMag=P_{99}-P_{1}, \end{array}$$ (8)

where *P*_*n*_ denotes *n*^th^ percentile of the activity. A penalty term *P*(*θ*) is also included in the objective for the estimation of the temperature parameters of the model from the epileptic discharge activity during cooling:

$$\begin{array}{c} P\left(\theta \right)=K\;({\left[\max\left\{v_{DC}\right\}-\max\left\{v_{BC}\right\}\right]}_{+}+{\left[\min\left\{v_{BC}\right\}-\min\left\{v_{DC}\right\}\right]}_{+}), \end{array}$$ (9)

where [⋅]_+_ = max{0, ⋅}, {*v*} is the simulated discharge activity centered with respect to the baseline, and K is penalty strength set to 1000. This term imposes the constraint that the range of discharge activity during cooling (DC) is contained within the range of the discharge activity before cooling (BC), that is, epileptic discharges are indeed suppressed during cooling. Since the model is stochastic, ten different simulations were taken for each set of parameters from which the MAPE is computed against the experimental data. Finally, after we are able to narrow down the parameter space to optimize the objective function, a global search is employed [53]. We used Dividing Rectangle (DiRect) method [54], a deterministic global optimization method that is less computationally expensive than stochastic evolutionary methods such as Genetic Algorithm which was used in [52]. Moreover, estimation was performed using a one-minute steady-state activity in contrast to dynamic estimation procedures such as Kalman Filtering [55] and Dynamic Causal Model [56].

A two-part estimation is performed for each experiment. The first part estimates the parameters of the Wendling model (no temperature-dependent parameters) that describes the activity of epileptic discharges before cooling. The second part estimates the temperature-dependent parameters (Q_10_ factors) during cooling using the result of the first part describing the pathological activity of the brain. Simultaneous estimation of all model parameters (before and during cooling) can be done, however, the two-part approach circumvents search issues in high-dimensional space. Furthermore, to address possible over-fitting, estimation of the Q_10_ values was done using the first 40 seconds of the one-minute activity during cooling. The next 20 seconds of the activity were used for validating the model estimates from which statistical tests are performed.

## Results

### Estimation of the parameters of the neural mass model from epileptic discharge activity before cooling

**Fig 6:**
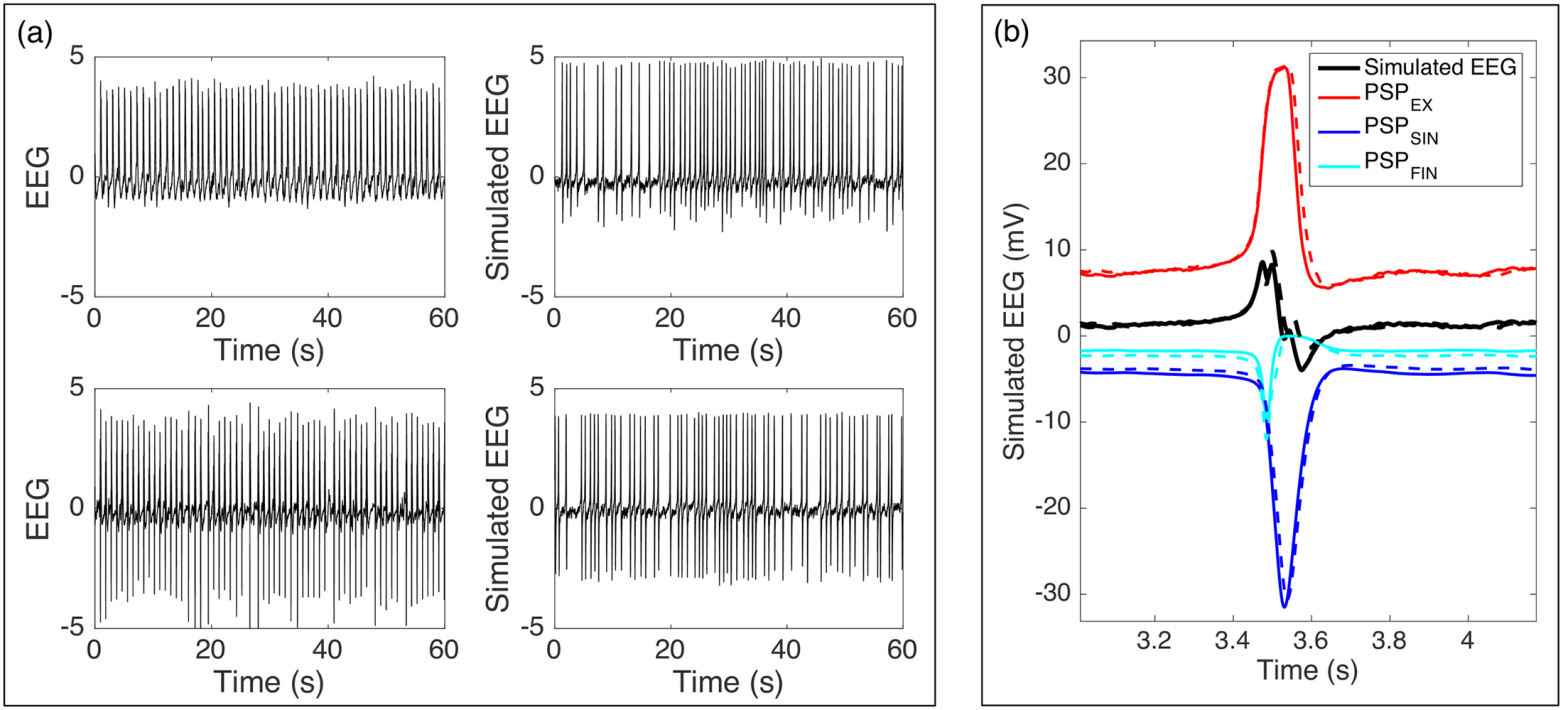
Estimation of Wendling model from activity before cooling. Wendling model captures several features of epileptic discharges before cooling such as average inter-discharge interval, magnitude and asymmetry of epileptic discharges. (a) Epileptic discharge activity before cooling (left) and simulated activity (right). Top plots are for rat 3 while bottom plots are for rat 4. (b) PSP from excitatory interneurons determine whether discharge activity tends to be symmetric (solid line) or asymmetric (broken lines) with respect to baseline.

It is generally accepted that epileptic activity results from changes in excitation-inhibition ratio. In the neural mass model, keeping the average excitation gain constant, excitation-to-inhibition ratio increases as *G*_*SIN*_ or *G*_*FIN*_ is decreased thereby simulating epileptic discharge activity. Exploration of the model shows that EEG recordings from the animal model of epilepsy used in the study is best explained by high average fast inhibitory gain *G*_*FIN*_ and low average slow inhibitory gain *G*_*SIN*_ (Table 2). This is consistent with previous findings that epileptic activity can arise when dendritic inhibition is impaired [34]. Fig 6(a) shows a reproduction of epileptic discharge activities before cooling for two of the five rats. We observe that lower values of *G*_*SIN*_ reproduce a discharge activity that is asymmetric with respect to baseline while higher values of *G*_*SIN*_ reproduce a discharge activity that tends to be symmetric with respect to baseline. On the other hand, increasing both *G*_*SIN*_ and *G*_*FIN*_ reduces the frequency of epileptic discharges by effectively reducing the average membrane potential of the primary cell population which is basically the simulated EEG.

**Table 2:** Estimated values of the parameters for the model of discharge activity before cooling.

rat	*G*_*SIN*_ (mV)	*G*_*FIN*_ (mV)
1	29.23	86.22
2	26.67	97.91
3	25.01	101.44
4	28.66	87.73
5	25.32	102.75

### Estimation of the temperature parameters of the model from epileptic discharge activity during cooling

**Fig 7:**
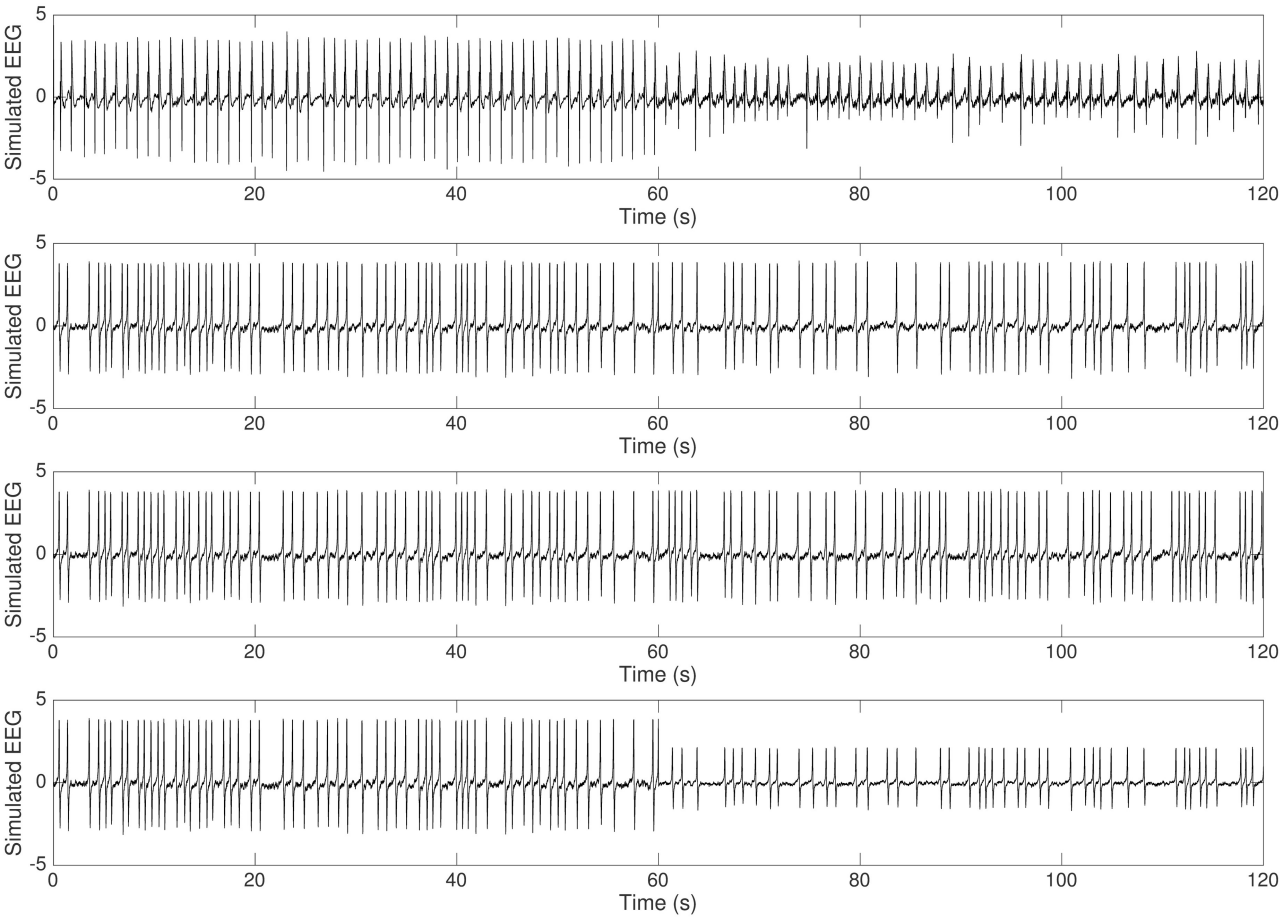
Effect of *Q*_10,*syn*_ and *Q*_10,*int*_. Synaptic or compensatory intrinsic mechanism alone does not capture suppression of epileptic discharges observed in experiment. (From top to bottom: Experimental Data, SYN, INT, SYN_INT.)

**Fig 8:**
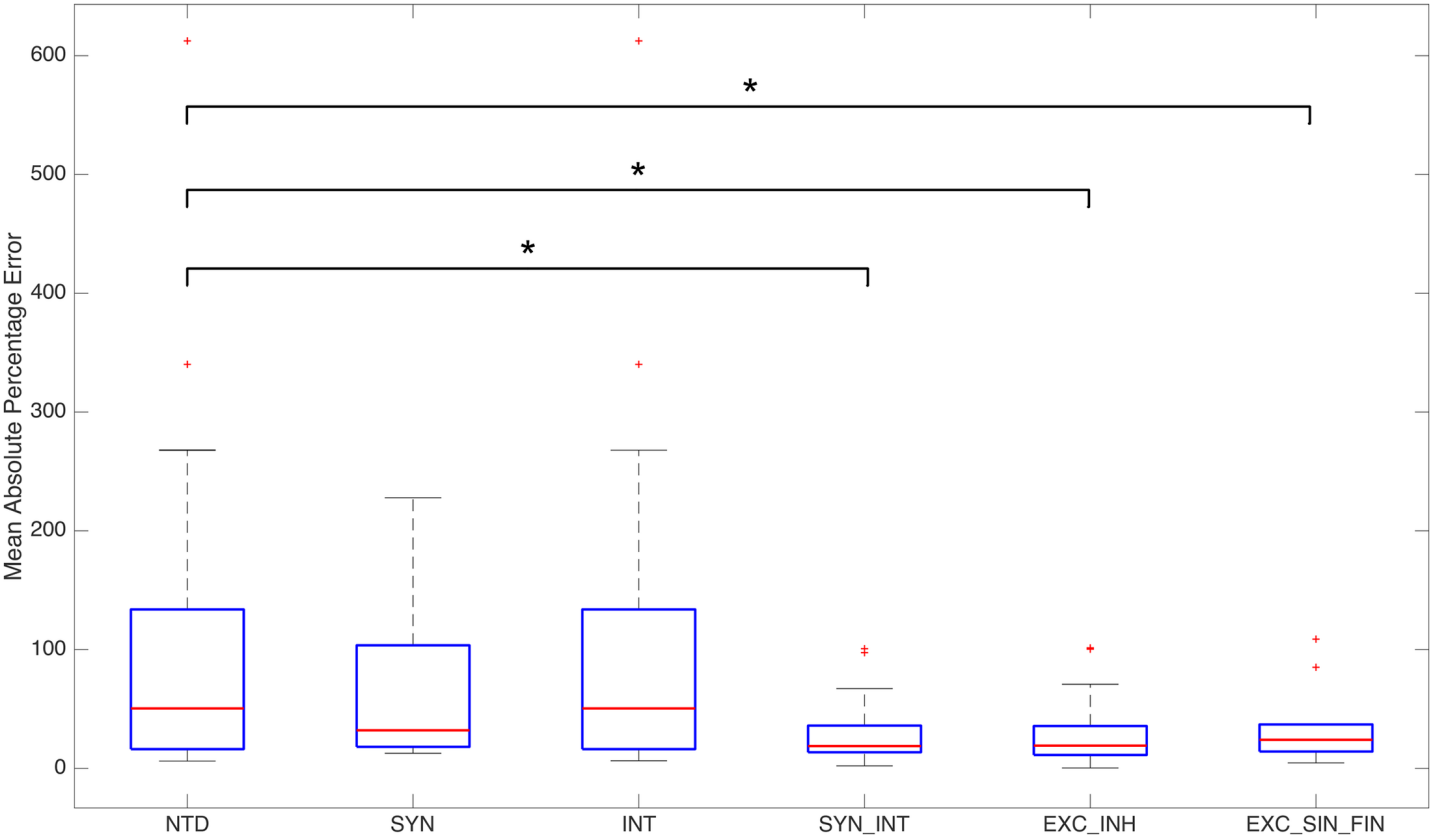
Comparison of models. Boxplot of MAPE of different models from experimental data. (*p < 0.01, Wilcoxon signed rank test)

The estimation of average slow inhibitory gain and fast inhibitory gain of Wendling et al. model was aimed to reproduce epileptic discharge activity recorded from the animal model of epilepsy used. Next, we estimate the parameters involved in the temperature dependence of the model from the activity during which focal cooling is applied in the epileptic brain area. To assess our temperature-dependent formulation, three models were estimated from the experimental data namely: SYN (synaptic mechanism only: estimate *Q*_10,*syn*_ with *Q*_10,*int*_ = 1.0), INT (intrinsic mechanism only: estimate *Q*_10,*int*_ with *Q*_10,*syn*_ = 1.0), and SYN_INT (synaptic and intrinsic mechanisms: estimate *Q*_10,*syn*_ and *Q*_10,*int*_). The results of the estimation were compared to no-temperature dependence (NTD) model (*Q*_10,*syn*_ = 1.0, *Q*_10,*syn*_ = 1.0). As discussed earlier, SYN captures changes in the frequency of epileptic discharges but not their magnitude (Fig 5). On the other hand, INT, as expected, yields estimates that are almost unity (like in the case of NTD) since the model does not have anything to compensate for having *Q*_10,*syn*_ = 1.0, i.e. no changes in average PSP yield no changes in the average firing rate. These suggest that temperature dependence in the post-synaptic impulse response function or firing response function alone does not capture the effect of cooling on the epileptic discharges (Fig 7). In fact, when both functions have temperature dependence as formulated (SYN_INT), we see that suppression of epileptic discharges is reproduced. Fig 8 shows the boxplots of the mean absolute percentage error (MAPE) of the different models from fifteen cooling experiments. Note that the MAPE are computed from the last twenty seconds of the epileptic discharge activity during cooling which is apart from that used for the estimation (see Materials and Methods). A Wilcoxon signed rank test shows that SYN_INT is significantly different from NTD model (*p* = 0.0034).

It is also interesting to look at the estimated values of *Q*_10,*syn*_ and *Q*_10,*int*_ using SYN_INT model (Table 3). We can clearly see that *Q*_10,*int*_ is only slightly less than *Q*_10,*syn*_. This is consistent in all estimations performed from experiments on five rats. We also performed estimation of Q_10_ factors from each cooling experiment per rat where we find cases in which *Q*_10,*int*_ is slightly greater than *Q*_10,*syn*_. These cases correspond to experiments where there are slight increases in the frequency of epileptic discharges during cooling. However, in the results that we present here, Q_10_ factors are estimated from three cooling experiments per rat which yield *Q*_10,*int*_ values that are all slightly less than *Q*_10,*syn*_.

**Table 3:** Estimated values of Q_10_ factors from three different models.

Model	*Q*_10,*syn*,*EX*_	*Q*_10,*syn*,*SIN*_	*Q*_10,*syn*,*FIN*_	*Q*_10,*int*_
3	1.9254			1.9108
	1.8375			1.8279
	1.7726			1.7634
	1.7273			1.7217
	1.0926			1.0925
4	1.8056	1.8549		1.7500
	1.8333	1.8219		1.8333
	1.8333	1.7963		1.8538
	1.8379	1.8219		1.8461
	1.1667	1.1665		1.1668
5	1.7222	1.5000	1.8457	1.8333
	1.6111	1.8004	1.1667	1.8333
	1.8333	1.7597	1.9774	1.7963
	1.8333	1.7958	1.8774	1.8333
	1.1666	1.1661	1.1670	1.1667

### Bifurcation of discharge activity with respect to Q_10_ factors

**Fig 9:**
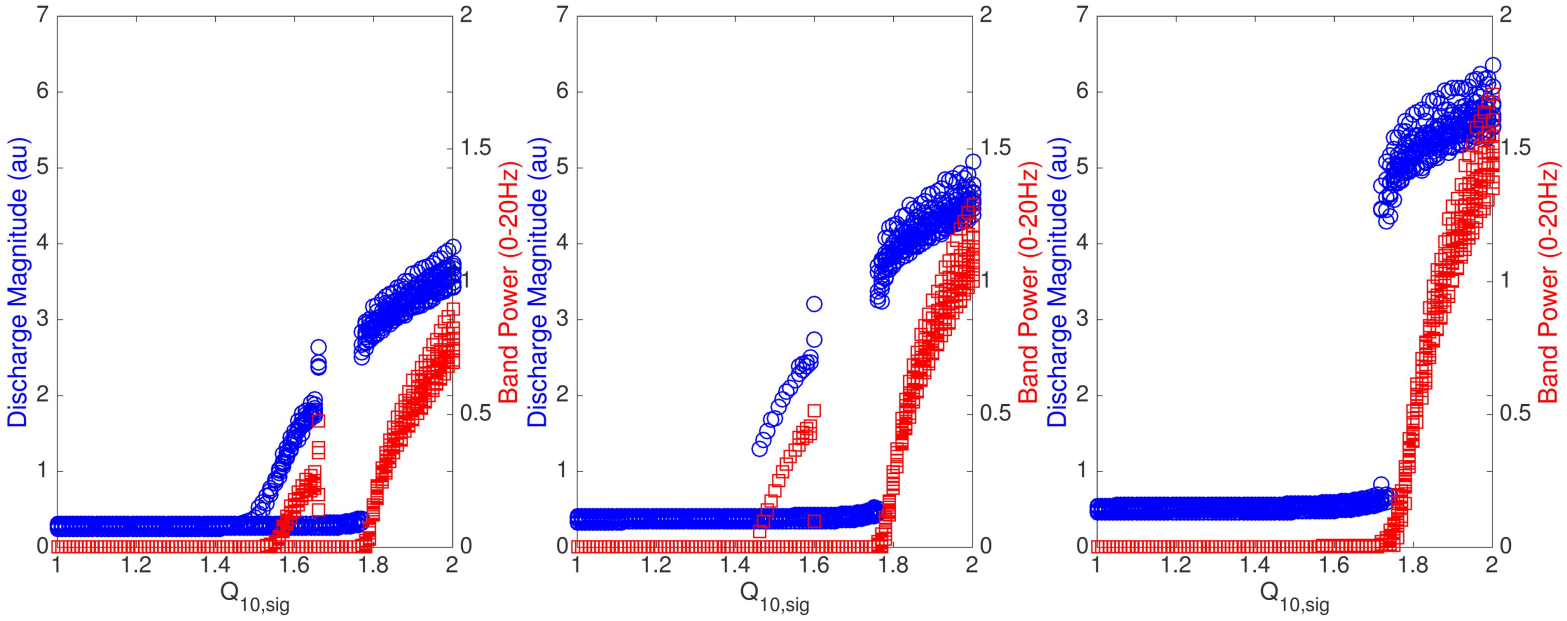
Bifurcation with respect to *Q*_10,*int*_. With *Q*_10,*syn*_ = 1.8, magnitude and frequency of discharges exhibit bifurcation behavior with respect to *Q*_10,*int*_ at different cooling temperatures. (From left to right: T = 15°C, 20°C, 25°C.)

**Fig 10:**
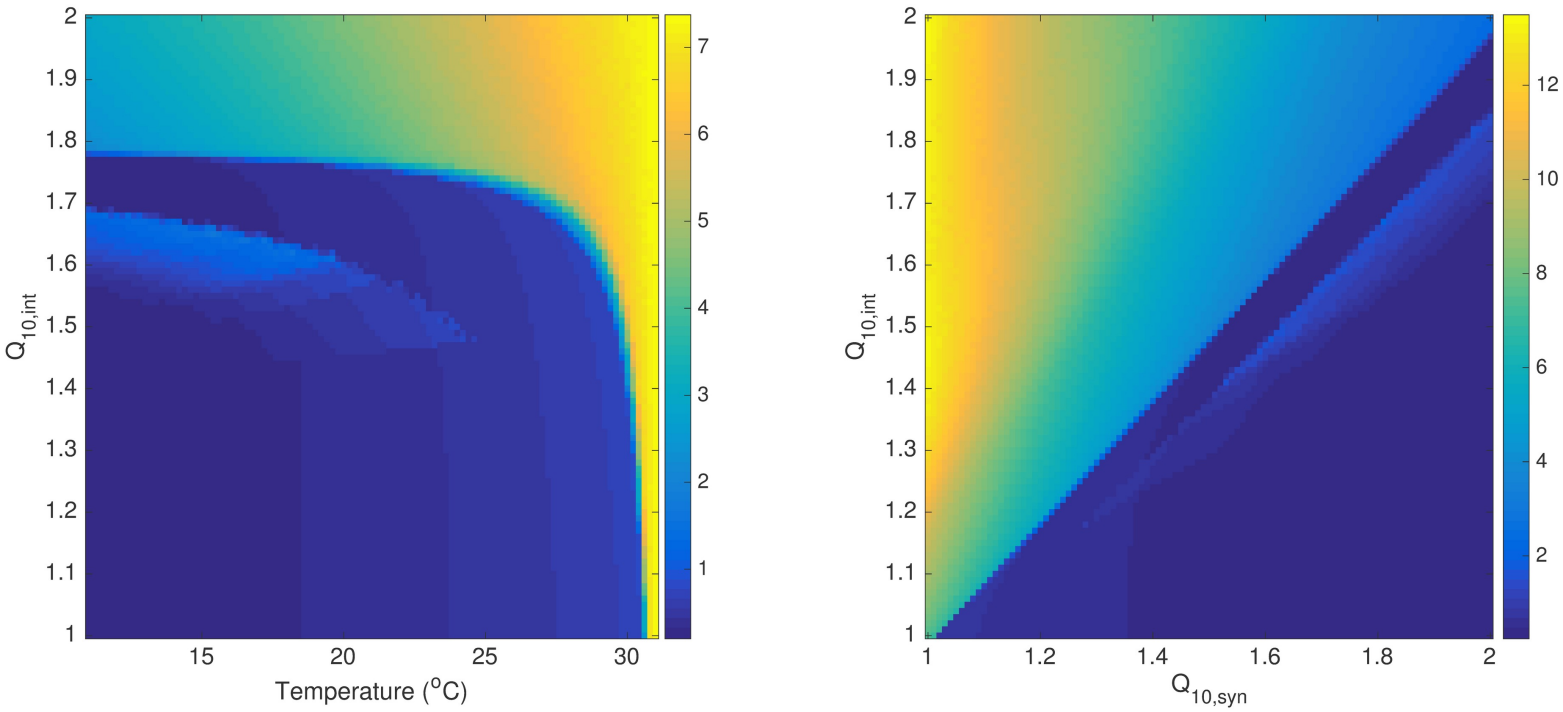
Bifurcation with respect to *Q*_10,*int*_ at different temperature and *Q*_10,*syn*_ values. Region of bistability with respect to *Q*_10,*int*_ vanishes at higher cooling temperatures (left). Similar bifurcation pattern is also observed at arbitrary *Q*_10,*syn*_ values with T = 15°C (right). The colorbars indicate discharge magnitude in arbitrary units.

Fixing *Q*_10,*syn*_ at 1.8, we vary *Q*_10,*int*_ from 1.0 to 2.0 at intervals of 0.01 and performed ten simulations of SYN_INT model with different random generator seeds. We find that the magnitude and frequency of simulated activity during cooling exhibit bifurcation behavior for different temperatures (Fig 9). There are three apparent bifurcation regions found for cooling temperatures 15°C and 20°C. From baseline activity, a bistable region occurs at around *Q*_10,*int*_ = 1.5 and vanishes at around *Q*_10,*int*_ = 1.66 going back to baseline activity until a sudden transition to discharge activity at around *Q*_10,*int*_ = 1.8 which is the same value at which *Q*_10,*syn*_ is fixed. The results of estimation from experiments lie around the third region where *Q*_10,*int*_ values are only slightly less than *Q*_10,*syn*_ values. This region corresponds to termination of epileptic discharges or suppression of epileptic discharges to a fixed magnitude. The bistable region, on the other hand, correspond to two possible activities depending on initial condition of the simulation- a baseline activity and an activity characterized by low-amplitude high frequency oscillations. This region, however, was not realized in the experiments. Hypothetically though, this suggests that seizure may occur with cooling when the compenstatory mechanism that involves the intrinsic excitability of neurons operates with *Q*_10,*int*_ values in this region. This bistable region vanishes at weaker cooling temperatures (Fig 10) indicating that such possibility of seizure may be prevented. Similar pattern of bifurcation is also observed with a bistability region that is wider at higher values of *Q*_10,*syn*_ and vanishes at lower values of *Q*_10,*syn*_ (Fig 10).

**Fig 11:**
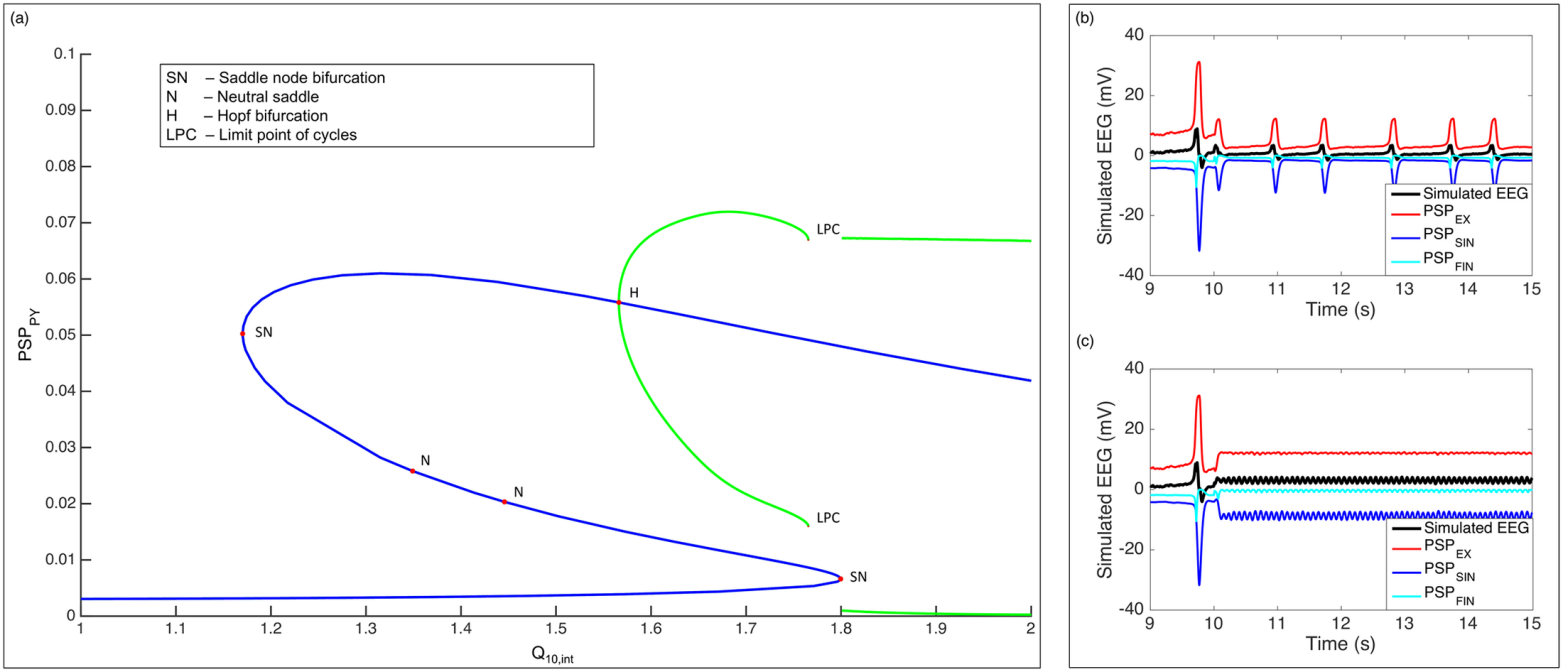
Insights on the observed bifurcation patterns of discharge activity during cooling (T = 15°C, *Q*_10,*syn*_ = 1.8). (a) Existence of a bistable region is confirmed using numerical continuation of noiseless version of the model. A Hopf bifurcation point within the bistable region indicates the possibility of seizure initiation at intermediate values of *Q*_10,*int*_. A limit point of cycles inside the bistable region explains transition from stationary activity (termination of seizures) to rhythmic discharge activity corresponding to suppressed discharge activity during cooling. (Green lines indicate maximum and minimum of limit cycles.) (b) PSP inputs from interneurons are attenuated resulting to suppression of discharge activity during cooling. (c) PSP inputs from interneurons exhibit low amplitude rhythmic activity contributing to initiation of seizure activity.

To gain more insight about the bifurcation behavior observed in the model, we performed a numerical continuation of the deterministic version of the model (standard deviation of input is zero) using MatCont [57]. Similarly, we fixed *Q*_10,*syn*_ at 1.8. Continuing from a fixed point with *Q*_10,*int*_ = 1.0, two saddle node bifurcations are found at around *Q*_10,*int*_ = 1.7996 and *Q*_10,*int*_ = 1.1702 (Fig 11(a)). From the second bifurcation point, a Hopf bifurcation is found at around *Q*_10,*int*_ = 1.566175 with negative first Lyapunov coefficient. This implies that a stable fixed point transitions into a stable limit cycle. These bifurcation points explain the observed bistable region in the original stochastic model above where low-amplitude high-frequency oscillations or a baseline activity can be observed depending on the initial state of the system. (Note that stationary state in the noiseless model corresponds to baseline activity in the stochastic model.) Furthermore, continuing from the Hopf bifurcation point, a limit point of cycles (LPC) is found at around *Q*_10,*int*_ = 1.68. A LPC is a saddle node bifurcation for periodic orbits where two limit cycles coalesce and annihilate each other. This explains the recovery of stationary state until the first bifurcation point at which the system exits the bistable region and goes back to stable periodic orbits (discharge activity). The transition point observed in the stochastic model (termination to suppression of discharge activity) is then a sudden jump from baseline activity resulting in a magnitude of suppressed discharge activity that is proportional to the width of the hysteresis loop for a particular temperature and does not gradually increase from the magnitude of a baseline activity. At weaker cooling temperatures, such bifurcation is not observed at least in the physiologically explicable region of Q_10_ values.

### Differential effect of temperature on PSP generation

**Fig 12:**
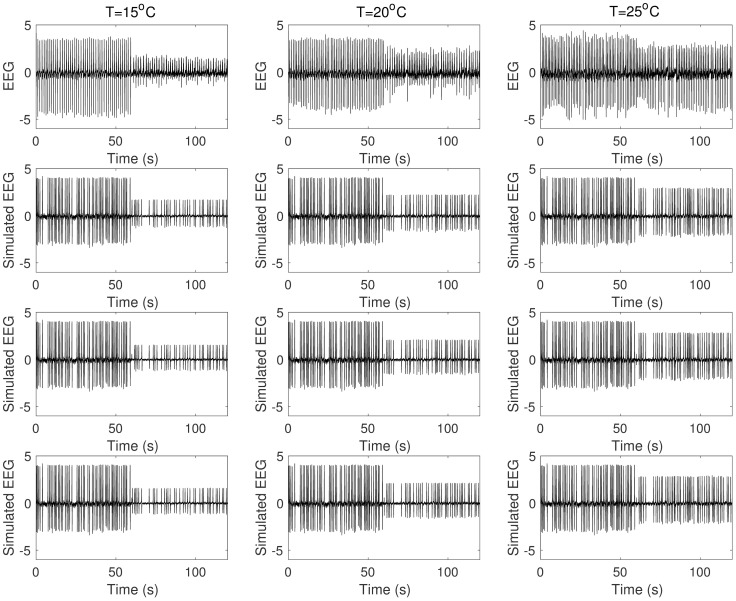
Simulated activity from rat 4. Suppression of epileptic discharges is replicated by the different models. (From top to bottom: Experimental data, SYN_INT, EXC_INH, EXC_SIN_FIN)

**Fig 13:**
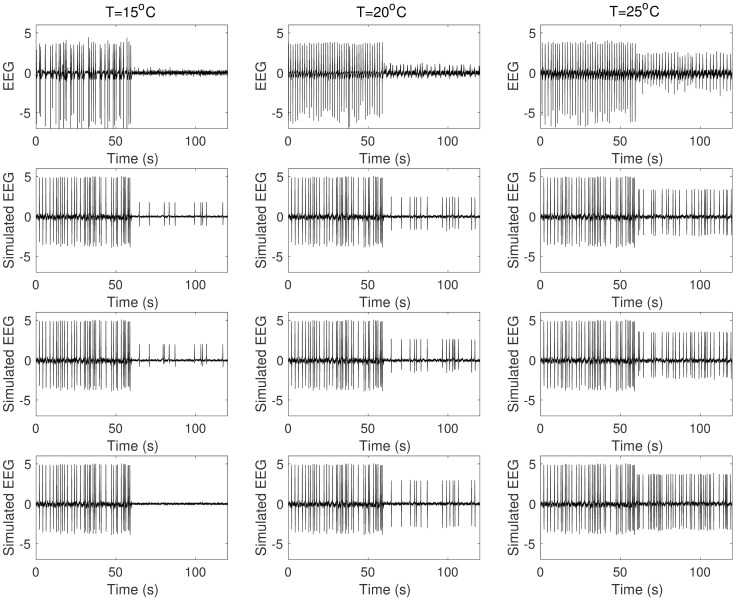
Simulated activity from rat 1. Termination of epileptic discharges at cooling temperature of 15°C is captured by model 5. (From top to bottom: Experimental data, SYN_INT, EXC_INH, EXC_SIN_FIN)

We also explored the possibility that cooling has differential effect on PSP generation of different neuronal populations. We investigate this by assuming that *Q*_10,*syn*_ is not homogeneous for different populations with different average synaptic gains. (*Q*_10,*int*_ is not differentiated across different subpopulations as we assumed that the temperature effect is the same across different populations in their intrinsic excitability mechanisms.) SYN_INT assumes homogeneous effect of cooling across different populations. Two more models were estimated to account for the possibility of such differential effect of cooling. In EXC_INH, we assume differential effect of cooling on excitatory and inhibitory PSP generation involving production of glutamate and GABA respectively. In EXC_SIN_FIN, we further assume differential effect of cooling on slow and fast inhibitory PSP generation involving slow GABA and fast GABA respectively. Estimation of these two models were also found to yield significant difference from NTD (*p* = 0.0034 and *p* = 0.0034 respectively). The two models however are not significantly different from SYN_INT (*p* > 0.01, Fig 8). It is interesting to note that EXC_SIN_FIN is able to capture termination of epileptic discharges from rat 1 under cooling temperature of 15°C which is roughly captured using SYN_INT or EXC_INH. Estimated Q_10_ values in Table 3 present some general observations. In EXC_INH model, *Q*_10,*syn*_ values are now slightly less than *Q*_10,*int*_ values except for rat 1 in which termination of epileptic discharges was observed. In EXC_SIN_FIN, higher *Q*_10,*syn*,*FIN*_ values were estimated especially with rats 3 and 4. On the other hand, lower *Q*_10,*syn*,*EX*_ values are observed for rats 1 and 2 in which termination of epileptic discharges were found while lower *Q*_10,*syn*,*SIN*_ values for rats 3 and 4 in which epileptic discharges are only suppressed during cooling. These observations suggest that termination or suppres sion of epileptic discharges can result from different synaptic responses of different neuronal populations to cooling. Figs 12 and 13 show how the different models reproduce termination or suppression of epileptic discharges in rats 4 and 1, respectively.

Finally, it can also be observed that the estimated Q_10_ values are between 1.7 and 2.0 except those estimated from rat 5 in which case the estimated values are less than 1.2. The estimation result from rat 5 can be substantiated by observing the activities during cooling of rat 5 at different temperatures showing less evidence of suppression of epileptic discharges (Fig 1).

## Discussion

Our study confirms the ability of Wendling et al. model to capture different brain activities particularly epileptic discharge activity induced in the animal model of epilepsy used. After a brute-force search in the *G*_*SIN*_ and *G*_*FIN*_ space (with *G*_*PY*_ = *G*_*EX*_ = 5.0), we find that the epileptic discharge activities from our animal model of epilepsy are best estimated in the range [24.0, 31.0] mV for *G*_*SIN*_ and [80.0, 110.0] mV for *G*_*FIN*_, the latter of which is not explored in the original model. Alternatively, we can keep *G*_*FIN*_ in physiological range [40.0, 60.0] mV but would entail that the number of synaptic connections from fast interneurons to pyramidal cells is twice than the standard value or that the maximum average firing rate of fast inhibitory interneurons is twice than that of the others (see Eq 3). This is still consistent with the findings of Wendling et al. [34] suggesting that impaired dendritic inhibition alters excitation-inhibition balance giving rise to rhythmic discharge activity capturing the effect of Penicillin G potassium in cortical tissues inhibiting GABA receptors [58]. Nevertheless, the estimated parameters indicate that our animal model of epilepsy can be best explained by much lower dendritic inhibition and much higher perisomatic inhibition compared to the standard range of values reported. High *G*_*SIN*_ values in fact supports [59] which reported high somatic inhibition together with impaired dendritic inhibition in experimental epilepsy. Meanwhile, asymmetric epileptic discharge activities with respect to baseline activity as seen from experiments with rat 3 can be reproduced with lower value of *G*_*SIN*_ (25.012 mV) and higher value of *G*_*SIN*_ (101.44 mV). On the other hand, symmetric discharge activity with respect to baseline is observed when dendritic inhibition is increased. Fig 6(b) illustrates that this symmtery (asymmetry) of the discharge activity (which is the summation of the PSP from excitatory and inhibitory interneuronns) is largely due to the PSP response of excitatory interneurons showing faster (slower) repolarization while the PSP responses of the inhibitory interneurons do not show significant changes.

Neurotransmitters play a central role in the generation of PSP [60]. They are released in response to Ca^2+^ influx after depolarization of pre-synaptic terminal and bind to their receptor molecules at the post-synaptic membrane opening or closing ion channels thereby generate excitatory or inhibitory PSP. It has long before suggested that neurotransmitter release has temperature dependence which causes changes in PSP generation [61]. This was confirmed by experimental observations of reduced efficacy of neurotransmitter vesicle release and reduced extracellular glutamate concentration during cooling [38, 40] that imply lower neurotransmitter concentration at the synapses to bind at the post-synaptic receptor and generate PSP. In light of this, it was straightforward to assume a temperature dependence on the post-synaptic impulse response function in a neural mass model. Similar to temperature-dependent formulation of Hodgkin-Huxley type neurons [62, 63], temperature dependence in Wendling et al. model is modelled using a temperature coefficient given by a Q_10_ factor. This factor accounts for mean-field effect of temperature to several processes occurring during PSP generation across the neuronal population. For example, diffusion of neurotransmitters, Ca^2+^, and receptor proteins [64–66] are slowed down at different rates at decreasing temperatures affecting efficacy of neurotransmitter vesicle release and the binding of neurotransmitters at the post-synaptic terminal receptors which regulate the activities of specific ion channels. Simply, *Q*_10,*syn*_ is added to the post-synaptic impulse response function and can be interpreted as direct attenuation of the average post-synaptic gain of the population or synaptic conductance of one neuron. This yields lower average PSP values when temperature is decreased from a baseline temperature. This decreases or increases the average membrane potential of the populations to which the population provides excitation or inhibition respectively. Reduced average membrane potential yields lower frequency of firing. In fact, we saw that termination of epileptic discharges results when the firing frequency approaches zero with *Q*_10,*syn*_ ≈ 1.085 with nonsignificant decrease in the magnitude of isolated discharges. In contrast, what was actually observed from experiments is that epileptic discharges are persistent during cooling but suppressed in magnitude. This is not reproduced by the model because of the nonlinearity of the firing response function. A Q_10_ value of 1.085 does not significantly suppress the magnitude of discharges but its effect on attenuated PSP responses significantly lowers the firing rate of the receiving population. Interestingly, in some cases in the experiment, slight increases in frequency of epileptic discharges were observed (Fig 1). These lead us to assume that a concomitant mechanism plays a role during cooling which may involve the intrinsic excitability mechanism of neurons compensating for the effect of reduced PSP on the average firing activity of the populations. Thus, a reciprocal Q_10_ factor was formulated as put forward in (Eq 10). Similarly, the Q_10_ factor involved here accounts for mean-field effect of temperature to several processes occurring during action potential generation such as diffusion of ions and ion channel gating across the neuronal population. Fig 4 shows how the average firing rate is compensated by the second temperature dependence. The firing frequency of a positive average membrane potential in the original firing response curve corresponds to an increased firing frequency at the same value of average membrane potential in the temperature dependent curve. The effect is opposite for negative average membrane potentials and rather minimal. A direct physiological interpretation of this mechanism can be examined if we write the equation in its equivalent form

$$\begin{array}{c} S\left(v\right)=\frac{2e_{0}}{1+e^{\left(\frac{Q_{10,int}^{(T-T_{0})/10}v_{th}-v}{Q_{10,int}^{(T-T_{0})/10}\sigma_{th}}\right)}}, \end{array}$$ (10)

where the Q_10_ factors are now with the parameters *v*_*th*_ and *σ*_*th*_. Recall that *v*_*th*_ is the average threshold of firing of neurons and *σ*_*th*_ is the variability in the thresholds of excitation of neurons. This then implies that as a compensatory mechanism, cooling lowers both the average and variance of the distribution of the firing thresholds of neurons in the population. Hence, even if the average PSP is reduced resulting in lower average membrane potential, epileptic discharges can still be persistent since lower average threshold of firing allows for subthreshold activity before cooling to become suprathreshold during cooling. This can be seen as a form of homeostasis in the firing activity of the neuronal population involving both synaptic and intrinsic excitability mechanisms. Surprisingly, the combined mechanisms result in suppression of epileptic discharges in terms of magnitude which is not captured if we assume temperature dependence in the post-synaptic impulse response function alone. This is because higher *Q*_10,*syn*_ values now significantly reduce PSP responses (Fig 11(b)) but the effect of which is compensated by the reciprocal of *Q*_10,*int*_. Slight increase in frequency of discharges observed in some of the experiments can be realized if *Q*_10,*int*_ is made slightly greater than *Q*_10,*syn*_. Further increasing *Q*_10,*int*_ proportionately increases the frequency of discharge activity (Fig 9).

Reduced threshold potential of firing during cooling has been reported on an early experiment with squid axons [27]. Experiments with mammalian brains [28, 67, 68] reported that cooling depolarizes cell membrane potential and increases input resistance. In [68], Volgushev et al. noted that cooling-induced depolarization of cell membrane occurs with an even higher gradient giving a marked decrease in the difference between the spiking threshold and the actual resting membrane potential. Thus, cooling brings the cells closer to spiking threshold, increasing excitability and decreasing variability in excitation levels across neuronal population. They proposed that such cooling-induced depolarization of the cell membrane may be attributed mainly by reduction of partial K^+^ conductance. Variability in threshold potential of firing has also been reported to increase with recent spiking activity [69]. We suppose that the opposite happens during cooling. As discussed earlier, cooling can decrease average firing rate of neurons which can imply less recent spiking activity. Henze and Buzsaki [69] suggested that prior action potentials cause Na^+^ channel inactivation that recovers with approximately a one-second time constant, increasing action potential threshold during this period. On the other hand, a study by Yu et al. [70] suggests that firing threshold variability can be explained by backpropagation of action potentials. Moreover, cooling was shown to strongly inhibit A-type K^+^ channels [71] in DRG neurons while these channels are reported to regulate action potential backpropagation in CA1 pyramidal neurons [72]. This might be in conflict with our finding that cooling reduces variability in firing thresholds since inhibited A-type K^+^ channels enhance backpropagating action potentials which in turn increases variability in firing thresholds. Then again, it is also possible that a net decrease in action potential backpropagation results as cooling can attenuate other critical factors such as density of Na^+^ at axon initial segment [73] and ion transport at nodes of Ranvier [74].

Estimation of the model from cooling experiments indicated that *Q*_10,*int*_ is only slightly less than *Q*_10,*syn*_. This means that during cooling, the intrinsic excitability mechanisms of neurons just balance out the effect of temperature change on PSP generation. At first, it seemed that when *Q*_10,*int*_ ≈ *Q*_10,*syn*_, discharge activity is suppressed but not terminated and when *Q*_10,*int*_ ⪇ *Q*_10,*syn*_, discharge activity is terminated. To verify this generalization, we simulated the model for different values of *Q*_10,*int*_ fixing *Q*_10,*syn*_ = 1.8. This led us to discover bifurcation patterns in the model which were confirmed using numerical continuation on the noiseless version of the model. First, we have verified that when *Q*_10,*int*_ ≈ *Q*_10,*syn*_, discharge activity is suppressed but not terminated. At this point, the intrinsic mechanism “fully” compensates the effect of the synaptic mechanism resulting to a discharge activity that has approximately the same frequency but reduced in magnitude (Fig 11(b)). However, we found out that when *Q*_10,*int*_ ⪇ *Q*_10,*syn*_, discharge activity is terminated only up to a certain value of *Q*_10,*int*_ and a seizure activity can arise with a wide range of intermediate *Q*_10,*int*_ values. As far as the authors are knowledgeable, there has been no report that seizure activity was ever observed in focal cooling of epileptic discharges. Moreover, *Q*_10,*int*_ values are not interpretable in terms of how intrinsic firing mechanisms can give rise to such values which would allow experiments to verify such finding. In theory, this should guide the design of implantable cooling devices which would necessitate a feedback control law to terminate cooling when a possible seizure can arise. Similar bifurcation patterns were observed for arbitrary values of *Q*_10,*syn*_ other than 1.8. Our estimation results indicated Q_10_ values around 1.8 which was, surprisingly, also reported in previous studies involving voltage-gated Na^+^ channel (VGNC) dynamics [13]. Then again, *in vitro* studies [68, 75] suggest that involvement of VGNC might be ruled out as abortion of epileptiform discharges were seen to be associated with a depolarization block. Perfect depolarization is against changes in the gating property as initially hypothesized, i.e., cooling is not inducing a liquid phase transition in phospholipid bilayer of the membrane thereby distorting the channel’s property, rather through other mechanisms.

Another interesting study by Motamedi et al. [75] with an *in vitro* epilepsy model showed that cooling has differential effect on the firing rates of pyramidal cells and interneurons. This actually motivated the models where we included more temperature dependent parameters to investigate possible differential effect of cooling on PSP generation. This relies on the assumption that cooling may have differential effect on different neurotransmitters responsible for generating PSP. However, in this study, the model parameters were estimated from *in vivo* EEG recordings which have clear departures from the aforementioned *in vitro* study. We can speculate though that it may be possible to reproduce such differential effect of cooling on the activity of pyramidal cells and inhibitory interneurons if we had isolated EEG recordings from pyramidal cell population and interneuronal population activities and from which we could estimate the model parameters with an appropriate modification of the objective function (Eq 9). Nevertheless, when the effect of cooling on inhibitory interneurons is much less than on excitatory interneurons, reduced average membrane potential of pyramidal cell population results and consequently, reduction in the average firing frequency of the population is observed as reported in the study.

The results presented in this paper only considered the steady-state effect and does not include transient dynamics of cooling on epileptic discharges although some experiments have noted the effect of rate of cooling on termination of epileptic discharges. For instance, an *in vitro* study [44] reported that during slow cooling, epileptic discharges persist with decreasing amplitude until termination is achieved with further temperature drop. In contrast, rapid cooling achieves immediate termination of the discharges. The gradual decrease in amplitude of epileptic discharges during slow cooling can be captured by the model using an appropriate model for temperature dynamics (e.g. Newton’s Law of Cooling). In its present form, immediate termination of discharges by rapid cooling can be explained by our model as a case where *Q*_10,*int*_ ⪇ *Q*_10,*syn*_, i.e. reduction in average and variance of firing thresholds across neuronal population is not able to compensate reduction in discharge frequency due to reduced average membrane potential resulting from attenuation of post-synaptic activity. Alternatively, such transient effect may be modeled by a Q_10_ that decays from a non-steady state value to a steady state value proportional to the rate of cooling. In most *in vitro* studies that we reviewed, steady-state termination of epileptic discharges was achieved using either slow or rapid cooling down to a constant temperature. In contrast, termination may not be always possible in *in vivo* setting. We surmise that the compensatory mechanism put forward by the model is more concomitant in *in vivo* than in *in vitro* environment.

Recent studies on epilepsy and epilepsy models have involved the role of non-neuronal cells such as astrocytes and microglia in mechanisms of seizure development such as reactive astrogliosis, glial-mediated inflammation, and Ca^2+^ signalling dysfunction [76, 77]. It may also be possible that cooling can attenuate activation of both neuronal and non-neuronal cells that will consequently impair their involvement in one or several hyperexcitability mechanisms. While there have been recent attempts at modelling the interaction of neuronal and non-neuronal cells [78, 79], formulation of temperature dependence on the models may involve multimodal recordings other than EEG (extracellular GABA and glutamate concentrations, cerebral blood flow) in focal brain cooling experiments to estimate the model parameters. This is an interesting direction which we hope to pursue in the future.
